# Alterations of Muscle Activation Pattern in Stroke Survivors during Obstacle Crossing

**DOI:** 10.3389/fneur.2017.00070

**Published:** 2017-03-03

**Authors:** Chenming Ma, Na Chen, Yurong Mao, Dongfeng Huang, Rong Song, Le Li

**Affiliations:** ^1^Department of Rehabilitation Medicine, Guangdong Engineering Technology Research Center for Rehabilitation Medicine and Clinical Translation, The First Affiliated Hospital, Sun Yat-sen University, Guangzhou, China; ^2^Key Laboratory of Sensing Technology and Biomedical Instrument of Guang Dong Province, School of Engineering, Sun Yat-sen University, Guangzhou, China

**Keywords:** stroke, obstacle crossing, electromyography, gait analysis, activation pattern

## Abstract

**Objective:**

This study investigates changes in the neuromuscular activation pattern of the lower limb muscles in stroke survivors when crossing obstacles of three different heights.

**Methods:**

Eight stroke survivors and eight age-, height-, and gender-matched healthy controls were recruited and instructed to cross over obstacles with heights of 10, 20, and 30% leg length. Surface electromyography (EMG) signals were recorded from the rectus femoris (RF), biceps femoris (BF), tibialis anterior (TA), and medial gastrocnemius (MG) of both limbs. Muscle activation signals were normalized to maximum voluntary contraction. Differences between groups and heights were compared using the root mean square of EMG, the cocontraction index of agonist and antagonist muscles, and power spectral analysis based on the mean power frequency (MPF). The correlations between the calculated variables and clinical scales such as Berg Balance Scale and Fugl-Meyer assessment (FMA) were also examined.

**Results:**

During the leading limb swing phase, the activation levels of all four muscles were greater in the stroke group than the healthy controls (*p* < 0.05), and the TA showed increased activation level with increasing obstacle height in both groups (*p* < 0.05). Cocontraction between the TA and MG was higher in the stroke group during the swing phase of the leading limb and between the RF and BF during the stance phase (*p* < 0.05). Similarly, for the trailing limb, increased cocontractions between the two pairs of agonist and antagonist muscles were found during the stance phase in the stroke group (*p* < 0.05). During the crossing stride, the frequency analysis showed significantly smaller MPF values in all four lower limb muscles in the leading limb of stroke survivors compared with healthy controls (*p* < 0.05). Moreover, significant correlations were found between the FMA scores and the BF and TA activations in the leading limb during the swing phase (*p* < 0.05).

**Conclusion:**

Greater activation levels of the lower limb muscles resulted in higher muscular demands for stroke survivors, which might lead to greater difficulty in maintaining balance. The increased cocontraction during obstacle crossing might be compensation for the affected stability and enable safe crossing for stroke survivors. The reduced MPF in the affected limb of the stroke group might be due to impairments in motor units or other complex neuromuscular alterations.

## Introduction

Stroke is a leading cause of disability associated with a loss of ability to generate force, which results in activity limitations and has a negative impact on motor function (1). Following a stroke, motor control impairments such as weakness, slow movements, spasticity, fatigue, and incoordination often occur in the lower limbs, which lead to gait abnormalities (2).

Daily walking commonly involves avoiding obstacles, such as doorsteps, stones, and stairs, and stepping across obstacles has been demonstrated to require greater muscle force, increased balance control, and enhanced muscle coordination than level walking (3, 4). One study showed that almost half of the tested stroke survivors failed to step across an obstacle, and their ability to maintain balance was compromised (5). The loss of balance in stroke survivors during obstacle crossing may lead to a high risk of falls and cause soft tissue injuries or fractures. Therefore, it is important to analyze the characteristics of the motion during obstacle crossing in stroke survivors. In spite of compromised balance, it is possible that stroke survivors may use compensatory strategies to avoid falls. It would therefore be helpful to understand the mechanism of preventing falls and ensuring safe crossing.

The balance and postural stability of stroke survivors have been quantified using kinematic and kinetic parameters, such as joint angles, joint moment, end-point distance, the distance between the center of mass and the center of pressure, and the ground reaction force (6–8). The balance was compromised in stroke survivors, and they might take a strategy involving greater pelvic posterior tilt and greater joint angles to ensure enough toe-obstacle clearance compared with healthy controls during obstacle crossing. However, little has been investigated about the neuromuscular changes during obstacle crossing. Electromyography (EMG) signals recorded from the surface of the muscles show the activity of motor neurons, which can reflect the relative level of muscle activation and provide valuable information about muscle function (9). Hahn et al. compared the EMG signals of the lower limb muscles between the elderly and the young (10). They found that elderly adults were able to negotiate different heights during walking and that the higher muscular demands could lead to a high risk of falls. The level of activity was reduced in the hemiparetic muscles in stroke survivors compared with the normal subjects, while the muscle’s activity of the non-paretic side was increased compared with the normal subjects, which helped maintain standing balance in response to sideways pushes (11). However, the neuromuscular mechanism of maintaining balance when crossing obstacles of different heights remains to be investigated in stroke survivors. Stepping over obstacles of different heights requires varying the activation levels of the agonist and antagonist muscles. Muscle cocontraction is the simultaneous activity of agonist and antagonist muscles (12, 13). Kitatani et al. demonstrated increased muscle coactivation in the trailing limb of stroke survivors with increasing obstacle heights, which could increase postural stability and decrease the rate of trips (14). However, they did not compare the coactivation patterns between healthy subjects and stroke survivors during obstacle crossing to understand more about the coordination mechanism following stroke.

Electromyography signals can also be analyzed in the frequency domain based on the power spectrum to reflect the neuromuscular function. The mean power frequency (MPF) and median frequency (MF) mainly reflect changes in the conduction velocity of the active motor units which could be damaged after stroke (15). It has been speculated that alternations of the EMG spectrum are related to loss of muscle fibers, changes in the composition of motor unit type (peripheral), synchronization of multiple motor units, and disorder control of motor unit (central factors). Many studies have investigated the relationship between muscle activation level and MPF and MF values for both healthy people and stroke survivors. Decreased MPF values are usually found in the muscles of the paretic side compared with the non-paretic side (16, 17). The amplitude of the EMG signals increases with the level of muscle force, but studies on the relationship between the EMG spectrum and contraction force remain uncertain. Hu et al. found slightly decreased MF values in the paretic biceps brachii with increased muscle contraction in stroke survivors (18), while Kaplanis et al. reported increased MF in the EMG of the biceps brachii with increased isometric torque in healthy subjects (19). EMG spectral analysis could help better understand the cause of neuromuscular changes in the stroke survivors during obstacle crossing, which has not been investigated in previous studies.

This study investigates neuromuscular changes in stroke survivors to maintain balance when crossing obstacles of different heights in comparison with healthy controls. We examined the relative muscle activation levels, the cocontraction of the agonist and antagonist muscles of the knee joint and ankle joint, and the power spectrum of the muscles, also their relationship with the clinical scales. The results may provide knowledge of the mechanism of motor control during obstacle crossing and information for designing fall prevention programs for rehabilitation following stroke.

## Materials and Methods

### Participants

Eight stroke survivors and eight healthy subjects matched by age, height, and gender were recruited in this study (Table 1). The inclusion criteria for the stroke survivors included (i) the occurrence of a first stroke with unilateral hemiparesis lesions confirmed by magnetic resonance imaging or computed tomography; (ii) an interval of at least 3 months post-stroke; (iii) the ability to step across an obstacle height of 30% leg length; and (iv) the ability to sign an informed consent form. This study was approved by the Ethics Committee of the First Affiliated Hospital of Sun Yat-sen University. All procedures were conducted according to the Declaration of Helsinki and all subjects provided written consent before the experiments. The motor function of stroke survivors was evaluated by an experienced physiotherapist based on the Berg Balance Scale (BBS) and Fugl-Meyer assessment (FMA) for lower extremities.

**Table 1:** **Basic characteristics of study subjects**.

Characteristic	Stroke group (*n* = 8)	Control group (*n* = 8)	*p*-Value
Age, years (mean ± SD)	58.88 ± 10.61	60.62 ± 8.33	0.445
Height, cm	167.33 ± 7.35	165.51 ± 5.92	0.296
Mass, kg	63.57 ± 7.68	61.67 ± 8.94	0.392
Gender	Male = 6, female = 2	Male = 6, female = 2	
Brain lesion side	4 Right and 4 left		
Duration post-stroke, months (range)	12.51 ± 11.22 (3–29)		
Berg test scores (range)	40.38 ± 6.94 (27–47)	56 ± 0 (56)	<0.001^a^
FMA scores (range)	23.12 ± 3.48 (18–28)	34 ± 0 (34)	<0.001^a^

*^a^Significant effect using an independent t-test*.

*FMA, Fugl-Meyer assessment scale of the motor function in paretic low-extremity (total score: 34)*.

### Apparatus

Circular silver–silver chloride (Ag–AgCl) electrodes with a diameter of 5 mm and inter-electrode distance of 20 mm were bilaterally attached to the bellies of the rectus femoris (RF), biceps femoris (BF), tibialis anterior (TA), and medial gastrocnemius (MG) of the lower limbs of the subjects. The muscle groups showed obvious changes when stepping over obstacles (20). Eight pre-amplified wireless transmission modules (DTS Noraxon, Scottsdale, AZ, USA) at a gain of 4,000 were linked with the electrodes to record EMG signals at a sample frequency of 1 kHz.

A total of 35 spherical 15-mm infrared-reflective markers were fastened to the subject’s whole body according to the Vicon Plug-In Gait marker placement. A 6-camera 3D motion analysis system (Vicon Motion Systems, Oxford, UK) recorded the marker positions at a sample frequency of 100 Hz. Two force plates (464 mm × 508 mm × 83 mm, AMTI, Watertown, MA, USA) at a sample frequency of 1 kHz were placed in the middle of the path with the obstacle between them. All the EMG, kinematic, and kinetic signals were recorded simultaneously and processed by the Vicon Nexus operating system.

### Procedure

Anthropometric characteristics were measured before the gait analysis (height, leg length, and bodyweight). Leg length was measured with scaled tape from the anterior superior iliac spine to the lateral malleolus and used to calculate the obstacle height of each individual. The electrodes and spherical markers were then attached to the corresponding locations on the subject. Before the electrode placement, the area around the muscles were shaved and cleaned with alcohol, and surgical tape was used as appropriate around the electrode and amplifier to obtain better EMG signals.

When the preparation was finished and the subjects had enough rest, the subjects were instructed to walk at a self-selected speed with bare feet along an 8-m walkway with a height-adjustable obstacle placed midway. The leading limb was defined as the first limb to cross the obstacle. The stroke subjects were instructed to use their affected leg as the leading limb of the obstacle crossing, and the healthy controls were instructed to use their dominant leg. The obstacle was set to three height conditions (10, 20, and 30% leg length). The three height conditions were performed in random order, and three trials of each height condition were recorded. Trials in which the subjects touched the obstacle were ignored and excluded from analysis. Prior to the trials, subjects visually and manually inspected the obstacle, and then practiced two to three trials according to the therapist’s instructions. Subjects were reminded to perform the task within their limits of safety and stop if they felt at risk. A therapist accompanied the subject and walked to the side to provide protection and assistance if required.

After all the trials were finished, the subject was asked to rest for a few minutes and then instructed to lay supine with the tested limb placed at 90° hip and knee flexion, and the other limb resting in neutral to perform maximum voluntary contraction (MVC) tests (21). Another experienced therapist held a hand-held dynamometer (MicroFET3, Hoggan Inc., UT, USA; with the precision of 0.4 N and range from 13 to 1,330 N) stably as a resistance at the corresponding position of the measured joint (22), and the subject used tested muscle group to push maximally against the hand-held dynamometer for about 5 s. To measure the MVC of the TA and MG, the hand-held dynamometer was placed proximally to metatarsophalangeal joints on dorsal and plantar surface of foot. To measure the MVC of the RF and BF, the hand-held dynamometer was placed proximally to ankle on anterior and posterior surface of leg (23). The subject performed 2–3 submaximum voluntary contractions before the MVC test began to become familiar with the test procedure. MVC test included knee flexion and extension, dorsiflexion, and plantar flexion, and the MVC was recorded three times for each muscle. During the MVC procedure, subjects were verbally encouraged to ensure maximal recruitment.

### Data Processing

For all MVC and gait trials, raw EMG signals were collected at 1 kHz, band-pass filtered through a fourth-order Butterworth filter with a bandwidth of 10–350 Hz, full-wave rectified, and low-pass filtered through a second-order Butterworth filter with a cut off frequency of 6 Hz. The root mean square was calculated during a particular phase of the gait. The filtered signals from the gait trials were then normalized to the MVC for each muscle to determine the relative activation levels. The calculation of the cocontraction index (CI) required two more steps with a linear envelope: (1) subtraction of the average resting EMG activity and (2) normalization to the maximum value of EMG activation in each muscle during the MVC tests (24). The CI value is given by

$$\mathrm{CI}\text{=}\frac{1}{T}\int_{T}A_{ij}\left(t\right)dt$$

where *A_ij_*(*t*) is the overlapping activity of EMG linear envelopes after subtraction and normalization for the agonist and antagonist muscles *i* and *j, T* is the length of the signal period. The CI value varies from 0 to 1. Zero means there is no overlapping of the two EMG envelopes, and 1 means the two muscles are fully activated to 100% MVC during the trial.

The MPF was calculated using the band-pass filtered signals (through a fourth-order Butterworth filter with a bandwidth of 10–350 Hz) for each time window (the stance, swing, or entire gait cycle). The MPF value is given by

$$\mathrm{MPF}=\frac{\int_{0}^{\infty }fP\left(f\right)df}{\int_{0}^{\infty }P\left(f\right)df}$$

where *P*(*f*) indicates the power intensity curve and *f* indicates the frequency. The kinematic data were filtered by a 20-Hz low-pass Butterworth filter. We considered the toe-off time to be when the toe marker was 2 mm off the ground and the heel-strike time as when the force platform received enough signals to make the measurement reliable (25). The gait cycle was then divided into a swing phase and stance phase for a single lower limb.

### Statistical Analysis

Descriptive statistics (mean values and SD) were calculated for all dependent variables. The normalized EMG activation, CI, and MPF values were subjected to a repeated-measures two-way (group: stroke and control × obstacle height: 10, 20, and 30% of leg length) analysis of variance (ANOVA). The ANOVA results were adjusted using a Bonferroni *post hoc* test. If there was an interaction between the two effects, then one-way ANOVA was separately performed for the group effect and height effect. Pearson product–moment correlations were used to examine the relationships between the calculated variables and clinical scales. The level of significance was set at an alpha level of 0.05. All statistical analyses were done using SPSS 19 statistical software.

## Results

All subjects were able to complete the tasks with three different obstacle heights and the MVC tasks. No incidents of falling were observed, and we discarded the trials in which the subjects touched the obstacle or received help from the therapist. Subjects did not indicate discomfort during the tasks, nor did they show any feelings of fatigue.

**Figure 1:**
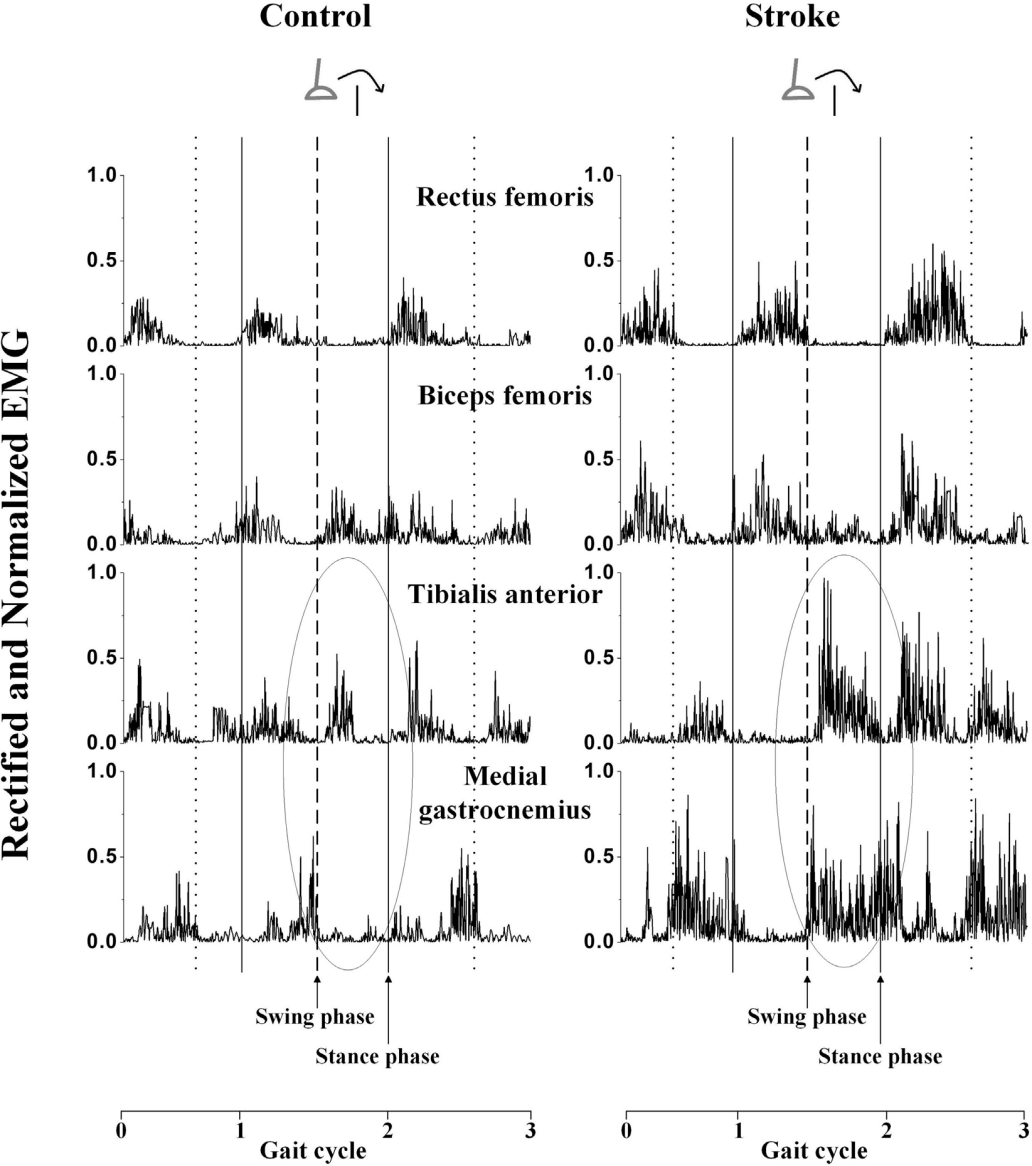
**The rectified and normalized electromyography (EMG) signals of the four muscles of the leading limb of a stroke survivor and a healthy control during 10% obstacle height**. The solid vertical lines indicate the time of foot contact, the dot vertical lines indicate the time of toe-off, and the dashed vertical lines indicate the time of toe-off when crossing the obstacle. Gait cycle 0–1 indicates the cycle before the obstacle, gait cycle 1–2 indicates the crossing cycle, and gait cycle 2–3 indicates the cycle after the obstacle. The circle labeled zone is used to demonstrate the phenomenon of cocontraction.

The rectified and normalized EMG signals showed that the muscles were activated at a corresponding phase during the gait in a typical trial of a stroke subject and control subject (Figure 1). The muscle activation level and activation duration were greater among the stroke survivors than the healthy controls in the four muscles of the leading limb respectively. Also, there were some abnormal cocontractions among stroke survivors. For example, the TA and MG (a pair of agonist and antagonist muscles) had greater coactivation level in the stroke survivor (CI = 12.15%) compared to the healthy control (CI = 4.31%) during the swing phase of the obstacle crossing, as indicated by a circle in Figure 1.

The stroke survivors showed greater relative activation levels in the leading and trailing limbs compared to healthy controls (Table 2). For all height conditions during the swing phase, the TA activation of the stroke survivors reached an average of 41% of their maximum capacity, in contrast to 29% in the healthy controls. Interactions were found between the groups and heights in the TA muscle of the leading limb during both swing and stance phases. The activation of the TA of the leading limb significantly increased with the obstacle height during both swing and stance phases (one-way ANOVA with *post hoc* tests, *p* < 0.05). The TA activation was significantly greater in the stroke survivors compared to the healthy controls at the 20 and 30% heights during the swing phase and at 30% height during the stance phase (one-way ANOVA, *p* < 0.05). For the trailing limb, the activation of the BF and TA also significantly increased with the obstacle height during the swing phase (*p* < 0.05).

**Table 2:** **Normalized electromyography activation percentages during swing and stance phases for rectus femoris (RF), biceps femoris (BF), tibialis anterior (TA), medial gastrocnemius (MG) of both leading and trailing limbs: mean (SD)**.

Limb/time	Muscle	Group	Obstacle height (% leg length)			
			10	20	30	Effect
Leading limb, swing phase	RF	Healthy	6.54 (4.06)	6.76 (4.02)	9.49 (8.02)	^a^
		Stroke	19.02 (18.44)	17.51 (16.30)	21.71 (19.68)	
	BF	Healthy	11.93 (5.96)	12.70 (4.51)	13.62 (5.48)	^a^
		Stroke	16.21 (9.67)	17.74 (7.92)	20.53 (11.25)	
	TA	Healthy	29.14^□^ (6.23)	30.14^d^ (6.82)	32.66^□,d^ (8.09)	^a,b,c^
		Stroke	34.03^Δ,□^ (10.49)	44.23^Δ,○,d^ (13.89)	56.38^□,○,d^ (16.78)	
	MG	Healthy	19.27 (4.99)	22.91 (7.13)	25.5 (10.47)	^a^
		Stroke	34.21 (11.00)	35.98 (17.42)	36.48 (22.5)	

Leading limb, stance phase	RF	Healthy	15.76 (10.52)	18.28 (11.23)	16.63 (12.10)	^a^
		Stroke	27.25 (14.68)	31.16 (16.53)	33.20 (14.24)	
	BF	Healthy	21.26 (7.06)	20.33 (5.55)	21.23 (3.89)	^a^
		Stroke	23.00 (11.49)	38.55 (12.71)	36.92 (12.47)	
	TA	Healthy	27.13 (12.80)	27.05 (16.72)	27.63^d^ (12.09)	^c^
		Stroke	27.24 (7.43)	28.88^○^ (11.41)	42.08^○,d^ (16.27)	
	MG	Healthy	27.21 (6.66)	29.77 (10.30)	34.05 (11.21)	^a,b^
		Stroke	27.75 (11.47)	44.05 (20.79)	42.11 (13.32)	

Trailing limb, stance phase	RF	Healthy	13.61 (8.39)	12.41 (6.91)	12.67 (8.45)	^a^
		Stroke	20.03 (12.42)	19.93 (11.8)	31.13 (26.62)	
	BF	Healthy	8.08 (4.49)	8.66 (3.40)	9.42 (4.03)	^a^
		Stroke	24.42 (11.26)	28.71 (17.22)	30.38 (14.39)	
	TA	Healthy	21.04 (10.31)	19.34 (7.02)	22.54 (12.12)	^a^
		Stroke	36.22 (15.25)	40.38 (11.29)	41.64 (18.34)	
	MG	Healthy	18.05 (5.94)	18.09 (7.58)	21.07 (8.71)	
		Stroke	28.16 (18.73)	27.94 (17.22)	28.84 (17.82)	

Trailing limb, swing phase	RF	Healthy	8.62 (2.53)	8.39 (2.78)	7.97 (2.77)	^a^
		Stroke	14.81 (5.92)	14.04 (4.87)	14.29 (5.44)	
	BF	Healthy	21.97 (8.98)	24.41 (9.57)	29.91 (13.69)	^b^
		Stroke	23.18 (11.51)	26.94 (15.68)	29.66 (14.08)	
	TA	Healthy	21.31 (6.74)	29.89 (7.17)	32.04 (5.54)	^a,b^
		Stroke	32.46 (12.23)	35.99 (12.05)	44.62 (16.74)	
	MG	Healthy	20.25 (8.44)	22.06 (6.78)	25.32 (9.61)	^a^
		Stroke	39.24 (7.73)	33.39 (10.31)	37.71 (12.61)	

*^a^Significant group effect*.

*^b^Significant height effect*.

*^c^Significant interaction effect*.

*^d^Significant group effect by post hoc test*.

*^Δ,□,○^Significant height effect by post hoc test. “Δ” means significant height effect between 10 and 20% leg length height. “□” means significant height effect between 10 and 30% leg length height. “○” means significant height effect between 20 and 30% leg length height*.

During the stance phase in the leading limb, the average CI for all height conditions of the RF and BF of the stroke survivors was 17.95 ± 7.90%, which was significantly greater than the average of 13.81 ± 4.89% (*p* < 0.05) for the healthy controls. Also, the average CI of the TA and MG was also significantly higher for the stroke survivors (10.53 ± 5.54%) than the healthy controls (7.60 ± 2.98%) during the swing phase (*p* < 0.05). Similarly, in the trailing limb, the average CI of the RF and BF (15.51 ± 5.18%) and the TA and MG (14.33 ± 8.52%) were significantly larger among stroke survivors than the healthy controls (RF and BF: 7.48 ± 2.39%, TA and MG: 7.85 ± 2.54%, *p* < 0.05), but only during the stance phase. The average CI showed no significant difference between stroke survivors and healthy controls in other conditions (*p* > 0.05).

**Figure 2:**
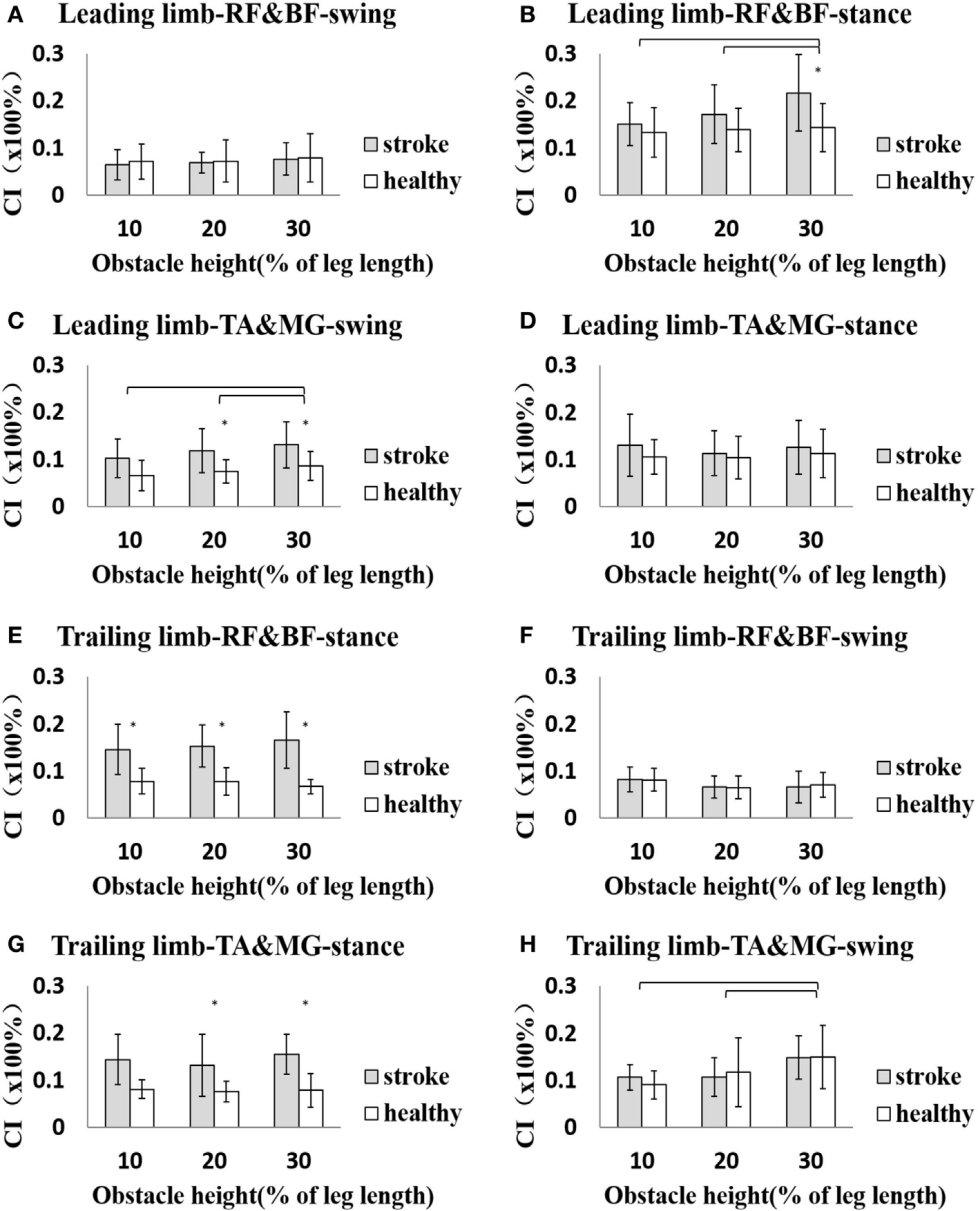
**The details of cocontraction index (CI) of each height for both leading and trailing limbs during swing and stance phases**. **(A)** The CI of rectus femoris (RF) and biceps femoris (BF) of leading limb during swing phase. **(B)** The CI of RF and BF of leading limb during stance phase. **(C)** The CI of tibialis anterior (TA) and medial gastrocnemius (MG) of leading limb during swing phase. **(D)** The CI of TA and MG of leading limb during stance phase. **(E)** The CI of RF and BF of trailing limb during stance phase. **(F)** The CI of RF and BF of trailing limb during swing phase. **(G)** The CI of TA and MG of trailing limb during stance phase. **(H)** The CI of TA and MG of trailing limb during swing phase. The asterisk (*) indicates significant effect between groups. The bar (-) indicates significant effect between heights.

Figure 2 shows the details of the CI at each obstacle height for both the leading and trailing limbs during the swing and stance phases. For the CI of the two muscle pairs of the leading limb, a between-group difference was found at the 30% height in the RF and BF and the 20 and 30% heights for the TA and MG (*p* < 0.05). There was a significant difference between the 10 and 30% and the 20 and 30% heights for the RF and BF during the stance phase, as well as between the 10 and 30% and the 20 and 30% heights for the TA and MG (*p* < 0.05). For the trailing limb, between-group differences in CI were found during the stance phase except for the TA and MG at the 10% height (*p* < 0.05). There was also a significant difference between the 10 and 30% heights and the 20 and 30% heights for the TA and MG during the swing phase (*p* < 0.05, Figure 2).

**Figure 3:**
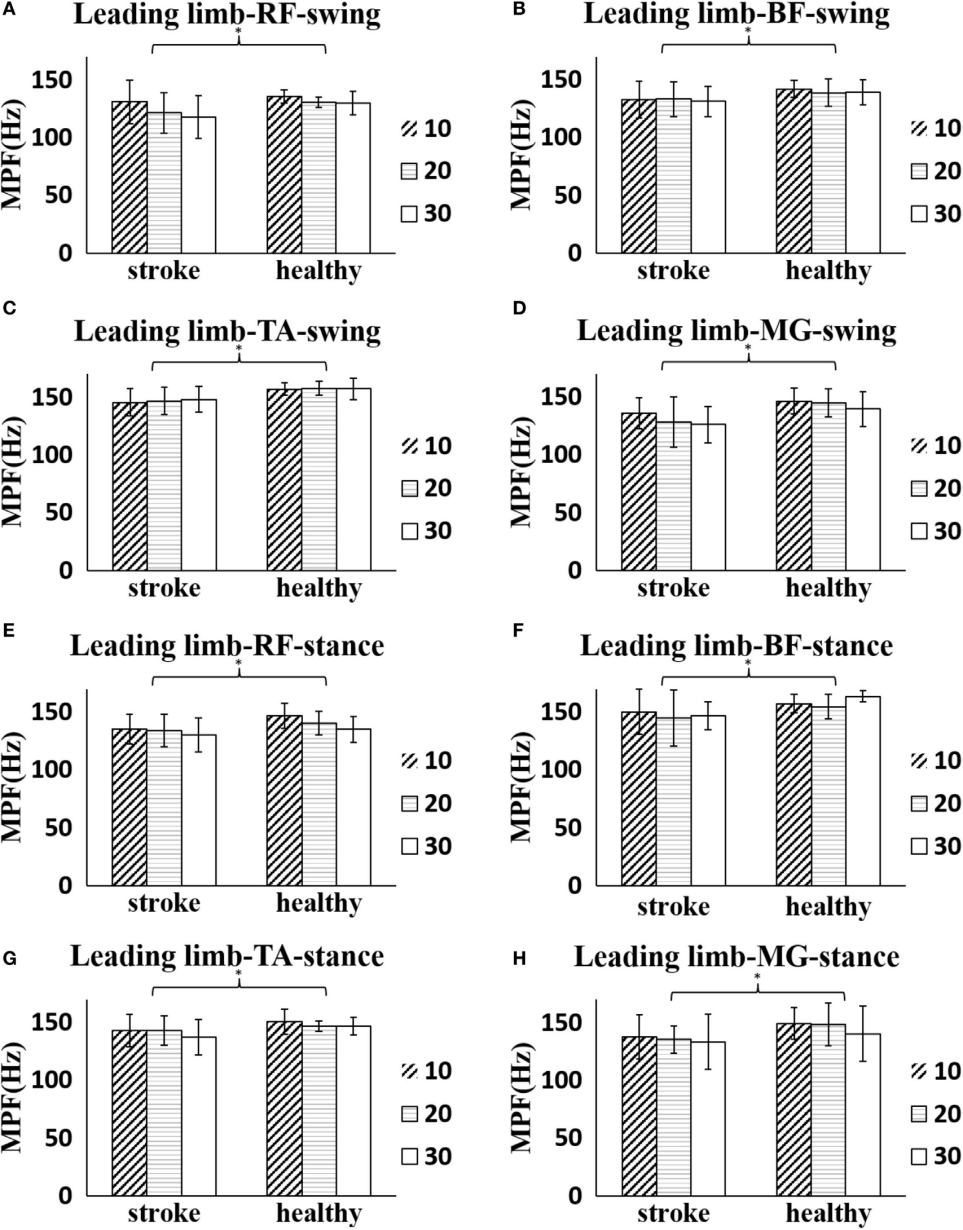
**The details of mean power frequency (MPF) value of four muscles of the leading limb of each height for both stroke survivors and healthy controls during obstacle crossing**. **(A)** The MPF of rectus femoris (RF) during swing phase. **(B)** The MPF of biceps femoris (BF) during swing phase. **(C)** The MPF of tibialis anterior (TA) during swing phase. **(D)** The MPF of medial gastrocnemius (MG) during swing phase. **(E)** The MPF of RF during stance phase. **(F)** The MPF of BF during stance phase. **(G)** The MPF of TA during stance phase. **(H)** The MPF of MG during stance phase. The asterisk (*) indicates significant effect between groups.

The stroke survivors had significantly smaller global MPF values of the leading limb during the entire crossing gait cycle (RF: 130.18 ± 13.33 Hz, BF: 134.67 ± 18.69 Hz, TA: 145.35 ± 12.52 Hz, MG: 130.18 ± 13.51 Hz) than the healthy controls (RF: 139.26 ± 10.21 Hz, BF: 148.40 ± 9.57 Hz, TA: 151.89 ± 6.96 Hz, MG: 140.18 ± 17.49 Hz), respectively (*p* < 0.05). However, no significant difference was found in the MPF values of the trailing limb during the entire crossing gait cycle between groups (*p* > 0.05). Figure 3 shows a more detailed comparison of MPF values in the leading limb at each obstacle height during different phases. The MPF of the four muscles of the leading limb was significantly smaller in the stroke survivors than the healthy controls (*p* < 0.05). There was also a decreasing trend (no significant difference) for the MPF value of the muscles as the obstacle height increased in most conditions. However, the MPF value of the TA in the leading limb showed an increasing trend (no significant difference) with increasing obstacle height.

**Figure 4:**
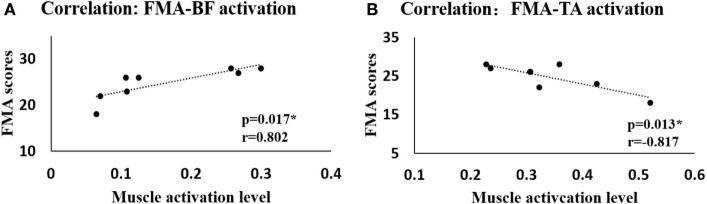
**The correlation between the Fugl-Meyer assessment (FMA) scores and the muscle activation levels when crossing the 10% leg length height obstacle**. **(A)** Correlation between FMA scores and muscle activation level of biceps femoris (BF). **(B)** Correlation between FMA scores and muscle activation level of tibialis anterior (TA).

We also examined the correlation between the calculated variables including activation levels, CI, and MPF values and the clinical scales (FMA and BBS). We only found a significant positive correlation between the activation of BF and FMA (*r* = 0.802, *p* = 0.017) and a significant negative correlation between the activation of TA and FMA (*r* = −0.817, *p* = 0.013) during the swing phase at the 10% obstacle height (Figure 4). No significant difference was found in other situations or between other variables and clinical scales.

## Discussion

In this study, we recorded the EMG signals of the four main lower limb muscles of stroke survivors and healthy controls during obstacle crossing. Our study demonstrated the activation levels of the muscles and the coactivation of agonist and antagonist muscles were greater, and the MPF values of the muscles were lower in the stroke survivors than the healthy controls, which indicated abnormal patterns of the gait and obstacle crossing following stroke. In addition, the significant correlations between the muscle activation of BF, TA, and FMA provided a reliable method to analyze the muscles of stroke survivors.

### Muscle Activation Level

When crossing the obstacles, the two groups encountered the same mechanical challenge (obstacle heights equal to the same percentage of leg length), but the stroke survivors showed greater overall muscle activation levels than the healthy controls in both the leading limb and the trailing limb (Table 2). Postural stability and the ability to maintain balance were impaired after stroke. Kirker et al. demonstrated that while a normal pattern of hemiparetic muscle activation was found in stepping, these muscles remained badly impaired in response to a perturbation and compensated by increased activity of the non-paretic muscles (11). Obstacle crossing challenges the stroke survivors to the limits of their capacity, and they are required to activate a greater level of their neuromuscular capacity to walk and safely step across obstacles. This may cause more serious postural instability (6). Similar results were also found in children with cerebral palsy. The RF and MG activation have been reported to increase in the swing phase of gait, which is considered an abnormal activation pattern that results from muscle weakness caused by cerebral palsy (26). According to Hahn et al., the relatively higher activation level might lead to muscular fatigue and place stroke survivors at higher risk of falls (10).

We found that the activation level of TA increased with increasing obstacle height in the swing phase of both leading and trailing limbs in stroke survivors. The TA muscle is the primary ankle dorsiflexor and is activated from the initial swing to let the ankle joint turn into a neutral position. However, Antonopoulos et al. found no significant height effect of the activation of the TA muscle for young adults during obstacle crossing (27). The increased activation level of TA in stroke may related to spasticity. Because of the excessive coactivation of the antagonist (MG), the increased activation level of TA was necessary to ensure a safe clearance during obstacle crossing.

### Muscle Cocontraction

Although cocontraction is inefficient for joint movement, it might be important for providing joint stability, especially in tasks like obstacle crossing (14). When the leading limb steps over an obstacle, the cocontraction of the TA and MG is greater in the stroke survivors compared with the healthy controls and also increased with increasing obstacle height (Figure 2C). The dorsiflexor (TA) strength is weakened after stroke, and the cocontraction of the antagonist (MG) might reduce the dorsiflexion range but increase the stability during the swing phase to ensure safe crossing (28). Also, the cocontraction of thigh muscles (BF and RF) is greater in the post-obstacle stance phase of the leading limb of the stroke survivors compared with the healthy controls, which is helpful to maintain balance by controlling the knee position during loading (29) (Figure 2B).

The greater cocontraction of the two pairs of lower limb muscles with increasing height also indicated muscle weakness on the paretic side of stroke survivors, who needed greater cocontraction to maintain balance when crossing obstacles of higher heights. Muscle cocontraction is also related to postural stability and dynamic strength in osteoarthritis (30), cerebral palsy (31), and Parkinson’s disease (32). The increased muscle cocontraction is a metabolically costly process (33), but it may help preserve some mobility in those with weakness (34).

When the trailing limb steps over the obstacle, the cocontraction of the TA and MG also showed a height effect, but there was no significant difference between groups during the swing phase (Figure 2H). This indicates that the cocontraction might be an adaptation strategy for both stroke survivors and healthy controls to increase postural stability. Both of the two pairs of lower limb muscles showed greater cocontraction in stroke survivors during the trailing limb stance phase (Figures 2E,G), which could be attributed to the need for greater stability and motor adaptation to the weakness of the affected leading limb to support body weight (35, 36). With motor recovery, the muscle strength could be increased and enable a more efficient strategy with decreased muscle cocontraction. Hu et al. investigated the motor function recovery process in cases of chronic stroke and found that CI values decreased during the recovery process as motor function improved (24). Assessing muscle cocontraction helps us to understand the coordination mechanism in stroke survivors and the adaptation strategy they use to ensure safe crossing.

### Analysis of Power Spectrum

The overall power spectrum analysis of all subjects indicated that stroke survivors had reduced MPF of the surface EMG in the leading limb compared with healthy controls (Figure 3). The reduction in MPF or MF on the paretic side has been report previously (16, 17). The decrease in MPF values of the paretic muscles could be due to the loss of muscle fibers and impairments in the motor unit following stroke. The firing rate has been demonstrated to be lower on the paretic side of stroke survivors compared both with the non-paretic side of stroke survivors and with the healthy controls during contraction (37, 38), which might also cause the reduced MPF values.

Alternations in the EMG spectrum have also been reported in children with cerebral palsy. Wakeling et al. and Gestel et al. found increased MPF value in such children compared with asymptomatic controls during gait and related it to the muscle dysfunction (26, 39). The assessment of the EMG spectrum could be used as an evaluation tool for functional muscle strength. No significant difference of MPF was found in the trailing limb between stroke survivors and healthy controls. This might be due to our instruction to the stroke survivors to use their affected side to cross the obstacle first and to use the unaffected limb as the trailing limb. This might have taken less effort to cross the obstacle and resulted in the lack of a significant between-group difference.

Obstacle crossing is a complex task, and the different heights place different demands on the subjects. Both groups showed a decreasing trend in MPF value with increasing obstacle height in the leading limb except for the MPF of the TA (Figure 3). Gabriel and Kamen also reported a significant decrease in the spectra with the force in the biceps brachii (40). With increased muscle activation level, there might be an increased degree of motor unit synchronization for the stroke survivors with the increasing obstacle height, which was likely to lead to reduction in the MPF values and fatigue (41, 42).

### Correlation

One interesting finding in our study is that there were significant correlations between calculated muscle activation levels of BF and TA in the leading limb and FMA scores measured by the therapists during swing phase at 10% leg length height (Figure 4) in stroke survivors. No significant correlation was found between muscle activation levels and FMA scores during 20 or 30% leg length height obstacle crossing in stroke survivors. Performance of stroke survivors was more disturbed during challenging task such as for obstacle crossing of higher height (43), and they couldn’t take good control of themselves which led to abnormal patterns and large variations within group when step across the obstacle. The activation level of the BF increased while that of the TA decreased with increasing obstacle height. Proximal control is more efficient than distal control in the lower limbs for stroke survivors to ensure safe obstacle crossing according to kinematic analysis (7). Our research showed through EMG analysis that stroke survivors with high FMA scores had greater BF activation, which controlled the hip joint and knee joint to elevate the toe. At the same time, the TA (which is related to ankle dorsiflexion) was abnormally activated to a larger degree in the face of fall risk among stroke survivors with lower FMA scores. Similar to Li et al., we found no significant correlation between the MPF values and clinical scales or between CI and clinical scales (16). Nevertheless, the significant correlation between the muscle activation levels and FMA scores means that it is reliable to use EMG signals to analyze the muscles of stroke survivors.

### Limitations

This study has several limitations. The recruited stroke survivors in this study had good function, so the results might not be useful for moderate or severe stroke survivors. We instructed the stroke survivors to use their hemiparetic side to take the first step over the obstacle and to use the unaffected side as the supporting limb, which is safer for them. We neglected situations using the other limb as the leading limb, which might lead to a minor difference in the results. Moreover, significant differences between stroke survivors and healthy controls should also be interpreted with caution considering the relatively small sample size. We plan to recruit more stroke survivors of different functional levels and to instruct them to use both limbs as the leading limb in future work to obtain a more comprehensive understanding of the neuromuscular changes.

### Conclusion

In this study, stroke survivors were recruited to step across obstacles of three different heights and compared with healthy controls to investigate motor control mechanisms that could not be reflected during level walking. Although the stroke survivors could safely step across the obstacles, they demonstrated abnormal motor control patterns, such as greater overall muscle activation level and larger cocontraction of the agonist and antagonist muscles. These might result in muscle fatigue, which would lead to a high risk of tripping and higher energy cost. The reduction in the MPF values of the paretic side of stroke survivors could be related to impairments of the motor unit or other complex neuromuscular alterations. The decreasing trend of the MPF values when crossing higher heights might due to greater motor unit synchronization, which could also lead to fatigue. The significant correlations between muscle activation levels and clinical scales provided a reliable method of analyzing the muscle functions of stroke survivors. These findings could help therapists to understand the neuromuscular changes following stroke and work out specific methods for rehabilitation of the lower limb muscles.

## Author Contributions

CM analyzed the data, interpreted the results, and wrote the draft of the manuscript. NC conducted the experiment and collected the data. YM and DH participated in the experiment and interpreted the results. RS participated in data analysis and interpretation and revised the manuscript. LL designed the study and performed all stages of the study including data collection, analysis, interpretation, and substantial revision of the manuscript. All the authors approved the final version of the manuscript.

## Conflict of Interest Statement

The authors declare that the research was conducted in the absence of any commercial or financial relationships that could be construed as a potential conflict of interest.
